# Annotations

**Published:** 1895-03-23

**Authors:** 


### Annotations.

### Commercialised Physic: A Protest.

Probably many medical readers are now having offered to them what has just been offered to the present writer—some debentures in a well-known company, which carry 6 per cent., and which are no doubt very sound securities. The company sells medical specialities; and any medical men who may purchase the debentures can help forward the sale of the specialities, and so make still more secure the excellent interest which the shares will bear. To ordinary persons the purchase of such shares would present itself as a good stroke of business, and the recommending of a medical speciality in which a physician has a proprietary interest would seem to be the most natural thing in the world. Offers like the one we are now discussing are becoming quite common. We believe that there is one medical speciality at least which is "run" entirely by medical men. There may soon be dozens. But, in spite of all this, we protest against such commercial transactions with all the energy in our power; we call upon all honest medical men to consider the question thoroughly; and we invite all classes of the public to resist, denounce, and, if possible, stamp out such commercialisation of physic. Is it etiquette or professional vanity which prompts our protest? In no sense. It is honest, single-eyed medicine which is at stake. At the bedside the doctor must have a single eye to the well-being of his patient in ordering food and physic. Such a thing as a side-glance at his own profit is dangerous, and ought to be felt to be criminal, and rendered impossible. We cannot, in a brief article like the present, set forth all the pros and cons of the case. But there are certain things, such as science, religion, and medicine, which cannot be commercialised without being destroyed. Let the profession and the public reason this thing out thoroughly, and it will be found that our protest is justified by necessity, and must be heeded.

### Dampness and Rheumatism.

Names are responsible for many errors. Few things are more generally accepted than the causative connection between dampness of air and soil and the prevalence of rheumatic fever. Yet the Milroy Lectures, delivered before the Royal College of Physicians by Dr. Newsholme, go far to show that dampness has nothing to do with it. Doubtless there is a rheumatism, a painful condition of joints, ligaments, and tendons, which is extremely susceptible to damp. There are hobbling old men who, by virtue of their malady, become trusty village storm-signals, and their malady is rheumatism; but that is a very different thing from rheumatic fever, and one can but opine that the application of this same word "rheumatic" to these very different conditions has had much to do with the prevailing notion that rheumatic fever also is caused by dampness of air or soil. For exactly the opposite seems to be the truth. Dr. Newsholme says that the amount of this disease varies more or less inversely with the rainfall, and that, comparing the yearly and quarterly rainfall at Greenwich with the incidence of rheumatic fever as shown by hospital statistics, it is found that a heavy rainfall is usually associated with a low amount, and a light rainfall with an excessive amount, of the disease, although no exact proportion was observed between them. Observations in regard to the relation between the level of the ground-water and the prevalence of the disease were even more at variance with what has been so commonly accepted. On the theory of dampness one would have expected to find rheumatism more prevalent at times when the subsoil water approached nearer to the surface of the ground. But again, just the opposite is the case. Although a lowering of the ground-water does not appear to be always accompanied by a high prevalence of rheumatism, it seems constantly true that an excessive prevalence never occurs when the ground-water is high. To some the suggestion that rheumatic fever occurs in epidemics, analogous to those of infectious disease, will come as a novelty; nevertheless, it seems to be the case, and there are many facts which point to the probability that, like them, rheumatic fever is an infective disease—not necessarily infective directly from man to man, but by way of the house, or the soil, or the surroundings—and that among the conditions favourable to its spread is that state of soil which accompanies a prolonged lowering of the ground-water—in other words, the exposure to air of a subsoil usually lying in water. We cannot pretend that the Milroy Lectures have entirely cleared up all that is doubtful as to the affinities of rheumatic fever, but they go very strongly to support the view that it is one of a series of ground-air diseases, and that for the maintenance of health much more care should be taken than is commonly done in the construction of houses to prevent the ground-air being drawn into our dwellings.

### Climate and Death-rate.

The death-rate in London and some provincial towns during the past few weeks has produced much searching of heart among doctors, whatever may have been its effects upon the laity. The psychologist approaches mental problems from two sides, from the standpoint of the individual man, and from the standpoint of mankind in the mass. So doing he reaches sounder conclusions than he could by the study of the individual alone. In like manner modern physic, which always at least aims at being scientific, must be tested by two criteria, by the results it produces in individuals, and by the general fruits it shows in the improvement or otherwise of the health of men and women in great masses. So judged, the medicine of London during the past few years does not shine with any very decided splendour. What is the cause of this? Is it that modern medicine, though more scientifically studied, is less successful as an art than the medicine of our predecessors? Or is it that there are certain causes of ill-health in the modern world which are themselves modern, and over which medicine—as medicine—can exercise little or no control? The latter, it is to be feared, is the true explanation. We have confined our sanitary exertions too much to the ground; too much to solid and fluid impurities. The atmosphere we have almost neglected. The result of this is that, with the enormous additions to the size of our large towns which have been made during the past few years, the climate in those towns and their suburbs has been ruined. London is fast becoming little better than a smoky "city of the plain." Very soon, unless some effort is made, its climate will be so debilitating, depressing, and life-endangering, that life in it will no longer be worth living. What striking testimony is borne to the truth of this contention by the death-rate of the last few weeks! Our smoke and our fog are our destruction. To get rid of our smoke would be to largely get rid of our fogs. Have we energy enough left in us to essay the mighty task; or must we continue to tolerate until we are all suffocated?

